# Plants Used as Antihypertensive

**DOI:** 10.1007/s13659-020-00281-x

**Published:** 2020-11-11

**Authors:** Tarawanti Verma, Manish Sinha, Nitin Bansal, Shyam Raj Yadav, Kamal Shah, Nagendra Singh Chauhan

**Affiliations:** 1 I.K. Gujral Punjab Technical University (IKGPTU), Jalandhar, Punjab India; 2 Laureate Institute of Pharmacy, Kathog, Jwalamukhi, Kangra, Himachal Pradesh India; 3 Department of Pharmacology, ASBASJSM College of Pharmacy, BELA, Ropar, Punjab India; 4 Department of Chemistry, S.P. Jain College (Veer Kunwar Singh University, Ara), Sasaram, Bihar India; 5 Institute of Pharmaceutical Research, GLA University, NH#2, Mathura, Uttar Pradesh 281406 India; 6 Drugs Testing Laboratory Avam Anusandhan Kendra, 1st Floor Govt. Ayurvedic Hospital Building, Govt. Ayurvedic College Campus G.E. Road, Raipur, Chhattisgarh 492010 India

**Keywords:** Hypertension, Antihypertensive herbs, Blood pressure, Vasodilatation, Herbal medicines, Blood pressure regulation

## Abstract

**Abstract:**

Hypertension is a critical health problem and worse other cardiovascular diseases. It is mainly of two types: Primary or essential hypertension and Secondary hypertension. Hypertension is the primary possibility feature for coronary heart disease, stroke and renal vascular disease. Herbal medicines have been used for millions of years for the management and treatment of hypertension with minimum side effects. Over aim to write this review is to collect information on the anti-hypertensive effects of natural herbs in animal studies and human involvement as well as to recapitulate the underlying mechanisms, from the bottom of cell culture and *ex-vivo* tissue data. According to WHO, natural herbs/shrubs are widely used in increasing order to treat almost all the ailments of the human body. Plants are the regular industrial units for the invention of chemical constituents, they used as immunity booster to enhance the natural capacity of the body to fight against different health problems as well as herbal medicines and food products also. Eighty percent population of the world (around 5.6 billion people) consume medicines from natural plants for major health concerns. This review provides a bird’s eye analysis primarily on the traditional utilization, phytochemical constituents and pharmacological values of medicinal herbs used to normalize hypertension *i.e. Hibiscus sabdariffa*, *Allium sativum, Andrographis paniculata, Apium graveolens, Bidenspilosa, Camellia sinensis, Coptis chinensis, Coriandrum sativum, Crataegus* spp., *Crocus sativus, Cymbopogon citrates, Nigella sativa, Panax ginseng,Salviaemiltiorrhizae, Zingiber officinale, Tribulus terrestris, Rauwolfiaserpentina, Terminalia arjuna *etc.

**Graphic Abstract:**

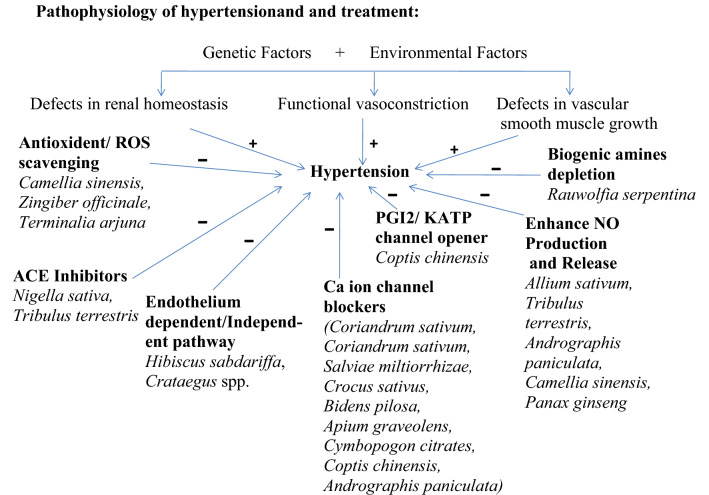

## Introduction

Hypertension is a serious medical condition and can increase the risk of heart, brain, kidney and other diseases. It is a major cause of premature death worldwide. Although several modern medicines are used to manage clinical hypertension but it is associated with various side effects. The use of natural herbal drugs with potential antihypertensive activity and fewer side effects can be a good substitute for synthetic drugs when associated with the change in lifestyle and light exercise.

**Fig. 1:**
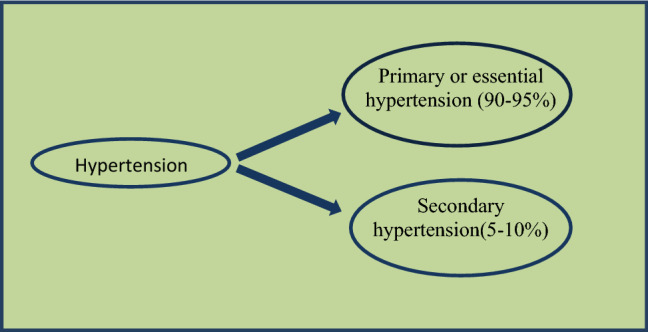
Types of hypertension

Blood pressure (BP) can be defined as the pressure exercise by blood inside the vessel walls. It is of two types: SBP (systolic blood pressure < 120 mmHg) and DBP (diastolic blood pressure < 80 mmHg). In Hypertension patient SBP increase upper than 140 mmHg or DBP elevateupperthan 90 mmHg. At present, 26.4% population of world suffered hypertension and it is predicted that in 2025 this rate would increase by 60%. Hypertension is mainly of two types (Fig. 1).

### Primary or Essential Hypertension (90–95%)

Patientshaveno clear identifiable cause which may contribute to elevation in blood pressure.

### Secondary Hypertension (5–10%)

Patients have mainly renal or adrenal disease as the root cause oftheir raised blood pressure [1].Other than this Factors like Nitric oxide NO and cardiac output and peripheral vascular resistance also play important role in hypertension [2, 3].Over aim to write this review is to collect information on the anti-hypertensive effects of natural herbs in animal studies and human involvement as well as to recapitulate the underlying mechanisms, from the bottom of cell culture and ex-vivo tissue data. According to WHO natural herbs/shrubs are widely used in increasing order to treat almost all the ailments of the human body, worldwide [4]. The type of phytochemical constituents present in any natural plant makes it useful to treat a particular ailment or group of ailments. The treatment with medicinal herbs/shrubs is essential and cheaper treatment with lesser rates of side effects as compared to allopathic treatment [5, 6]. Hypertension is a critical health problem and worse other cardiovascular diseases. Diuretics, alone or with other antihypertensive agents are in use regularly, to decline increased blood pressure by decreasing blood volume at the cost of dangerous and undesirable side effects. Interestingly, the use of drugs from natural sources as alternativesis the best choice for the treatment of hypertension and other diseases related to it [7]. Dubick explained that Plants are the regular industrial units for the invention of chemical constituents, they used as an immunity booster to enhance the natural capacity of the body to fight against different health problems as well as herbal medicines and food products also. In cultural, religious and folk traditions, herbal plants are explained as curative remedies for almost all of the ailments [8–10]. Since 1970, native plant medicines are also included in World Health Organization policies mainly for developing countries of the world. According to the United Nations World Health Organization, 80% population of the world (around 5.6 billion people) consumes medicines from natural plants for major health concerns [11, 12]. This review provides a bird’s eye analysis primarily on the traditional utilization, phytochemical constituents and pharmacological values of medicinal herbs used to normalize hypertension [13, 14].

## Mechanisms/Pathophysiology of Hypertension

### Blood Pressure Regulation

Several parameters like cardiac output, blood volume, the balance of arterial tone etc*.* of the cardiovascular system can conclude BP. The maintenance of physiological BP levels involves a multifaceted relationship of diverse elements of an incorporated neurohumoral system which includes the natriuretic peptides, renin–angiotensin–aldosterone system (RAAS), endothelium cells, immune system and sympathetic nervous system (SNS). Any imbalance in components of this incorporated neurohumoral system can indirectly or directly cause an increase or decrease in theaverage BP level. Moreover, if this imbalance remains for a long time, leads to damage of thetarget-organ (as CKD and left ventricular hypertrophy) and CVD also.

**Fig. 2:**
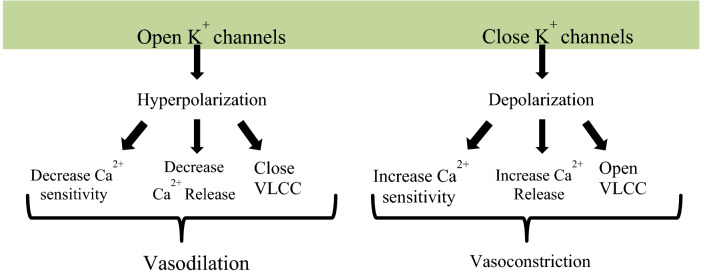
Effect of potassium channels blood vascular system

**Fig. 3:**
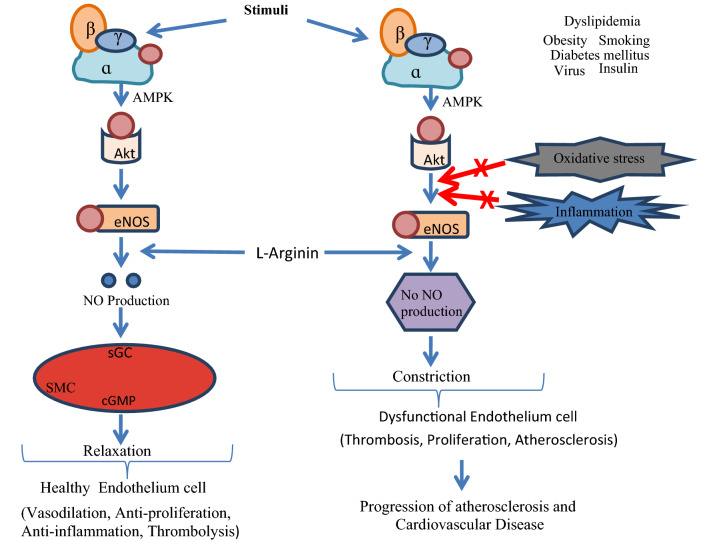
Oxidative stress and role of NOS on blood vascular system

**Fig. 4:**
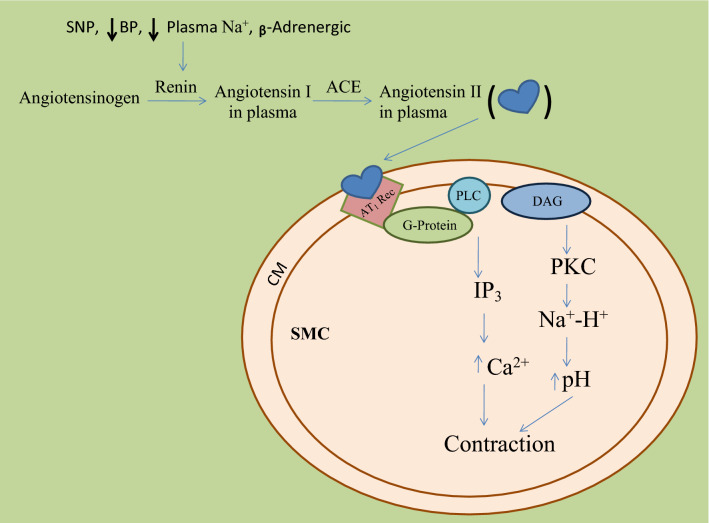
Role of ACE in blood pressure regulation. *BP* blood pressure, *PLC* phospholipase C, *SNP* single-nucleotide polymorphism, *ACE* angiotensin-converting enzyme, *DAG* diacylglycerol, *IP*_*3*_ inositoltriphosphate

**Fig. 5:**
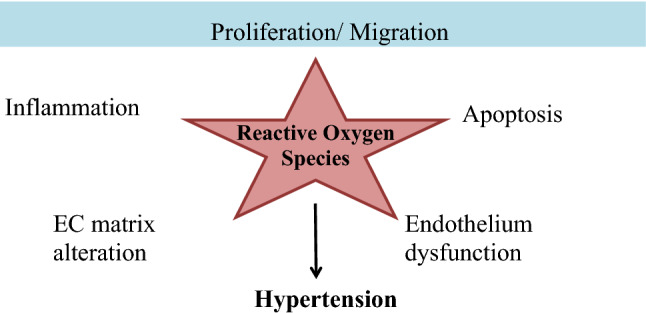
Effect of reactive oxygen species on blood vascular system

**Fig. 6:**
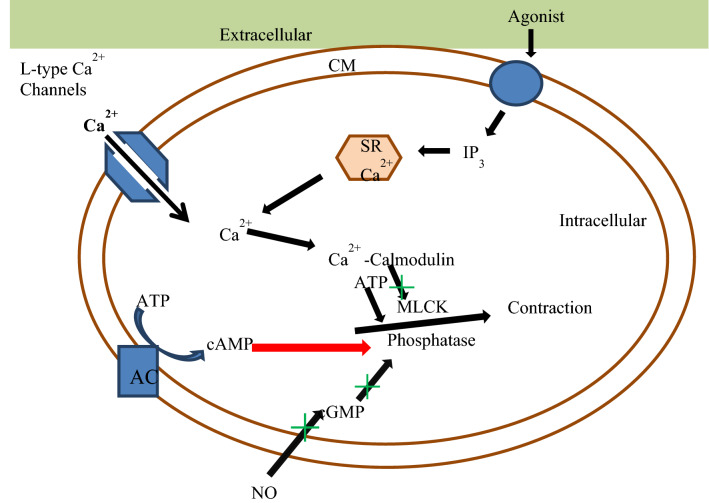
Mechanism of calcium channel mediated vasoconstriction. *CM* cell membrane, *SR* sarcoplasmic reticulum, *MLCK* Myosin light-chain kinase, *ATP* adenosine triphosphate, *AMP* adenosine monophosphate, *GMP* guanosine monophosphate, *NO* nitrous oxide, *AC* adenylate cyclase

Different Physiological effectors like Potassium channels [15] (Fig. 2), Nitric oxide (NO) (Fig. 3), renin angiotensin system (Fig. 4), Reactive oxygen species (Fig. 5)and Calcium ions (Fig. 6) modulate the vascular tone and any imbalance in these factors may lead to hypertension.

Nature has provided or inspired so many lead molecules that can effectively modulate these factors Tables 1 and 2. The following section comprises the details of common traditional herbs having a potential antihypertensive effect.

**Table 1:** Commonly used medicinal plants with antihypertensive activity

S. no	Medicinal herb	Experimental model used	Mechanism of action	Activity	References
1	*Allium sativum*	Fructose-fed rats	Inhibits ACE	Vasorelaxant	[40]
		Pulmonary arteries isolated from rat	Increases NO	Vasorelaxant	[37]
		Endothelial cells Human umbilical vein	Increases NO	Vasorelaxant	[38]
		Sprague–Dawley rat aortic rings	Increases H_2_S	Vasorelaxant	[39]
		VSMCs isolated from SHR	Reduce ang-II- enhanced cell cycle progression	Anti-proliferative	[43]
		Fructose-fed rats	Reduces NADPH activity	Antioxidant	[77]
		Human neutrophils	ROS scavenging	Antioxidant	[78]
		2 K-1C rats	Affects ant oxidation	Antioxidant	[79]
		Fructose-fed Wistar rats	Increases eNOS	Vasorelaxant	[77]
		Fructose-fed Wistar rats	Reduces VCAM-1	Anti-inflammatory	[77]
		Thoracic aortic VSMCs of sprague–Dawley rat	Induces Cx43 expression	Anti-proliferative	[80]
		High fructose-fed rats	Inhibits NF-κB	Anti-inflammatory	[81]
2	*Andrographis paniculata*	SHR	Scavenges ROS	Antioxidant	[49]
		SHR	Reduces ACE	Vasorelaxant	[49]
		Sprague–Dawley rats isolated hearts	Increases NO	Vasorelaxant	[44]
		Sprague–Dawley rats isolated hearts Sprague–Dawley rats	Blocks Ca^2+^ channels	Vasorelaxant	[44]
		Npr1 gene-knockout mice	Inhibits NF-κB	Anti-inflammatory	[82]
3	*Apium graveolens*	Rat isolated aortic rings	Blocks Ca^2+^ channels	Vasorelaxant	[58]
		CCl_4_-treated mice	Amplify antioxidants	Antioxidant	[59]
4	*Camellia sinensis*	Coronary heart patients’s brachial arteries	Increases flow-mediated dilation (FMD)	Vasorelaxant	[83]
		Coronary heart patients’s brachial arteries	Increases flow-mediated dilation (FMD)	Vasorelaxant	[84]
		Superoxide-generating system	Scavenges ROS	Antioxidant	[85]
		In vitro endothelial cells	Reduces VCAM-1	Anti-inflammatory	[86]
		Human endothelial cells	Inhibits NF-κB	Anti-inflammatory	[73]
		STZ fed SHR	Decreases NADPH oxidase	Antioxidant	[87]
		Strong man smokers (preclinical pilot)	Increases NO	Vasorelaxant	[72]
		Diabetic SHR	Inhibits eNOS uncoupling	Vasorelaxant	[88]
		Obese, hypertensive humans	Decreases TNF-α	Anti-inflammatory	[89]
		STZ-fed Sprague–Dawley rats	BlocksAT_1_ receptor	Vasorelaxant	[90]
		Sprague–Dawley rats nourished with streptozotocin (STZ)	Increases antioxidants	antioxidant	[90]
		STZ fed SHR	Hampered eNOS separation	Antioxidant	[88]
		C57BL/6 mice	Amplify antioxidants	Antioxidant	[91]
		Human aortic smooth muscle cells	Increases HO-1 enzyme	Anti-proliferative	[92]
5	*Coptis chinensis*	Rat aortic endothelial cells	Decreases NF-κB	Anti-inflammatory	[93]
		Rat aortic endothelial cells	Inhibits VCAM-1	Anti-inflammatory	[93]
		Hale and hearty humans	Decreases EMP	Vasorelaxant	[94]
		Cardiomyocytes of rat (hypertrophy stimulated by insulin)	Upregulates eNOS expression	Vasorelaxant	[95]
		CIHH rats’s thoracic aorta rings	Upregulates eNOS expression	Vasorelaxant	[95]
		CIHH rats’s thoracic aorta rings	Blocks Ca^2+^ channels	Vasorelaxant	[95]
		Cardiomyocytes of rat (hypertrophy stimulated by insulin)	Inhibits cardiac hypertrophy	Anti-proliferative	[95]
		Wistar rats with atherosclerotic renovascular sickness (ARD)	Increases antioxidants	Antioxidant	[96]
		ARD Wistar rats	Declined NADPH oxidase	Antioxidant	[96]
		Atherosclerotic renovascular rats	Decreases NF-κB	Anti-inflammatory	[96]
6	*Coriandrum sativum*	CCl_4_- induced hepatotoxicityin Wistar albino rats	Increases antioxidants	Antioxidant	[97]
		Decreases *NF-κB*	LPS-stimulated RAW 264.7	Anti-inflammatory	[98]
		Male Wistar rats with cardiotoxicity induced by isoproterenol	Inactivation of ROS production by b-adrenoceptor stimulation	Antioxidant	[99]
7	*Crataegus* spp.	Hypertensive rats (Induced by L-NAME)	Activates eNOS	Vasorelaxant	[100]
		Aortic rings of male Wistar rat	Activates eNOS	Vasorelaxant	[101]
		Mammalian arterial rings	Activates eNOS	Vasorelaxant	[101]
		Enzymatic assay	Scavenges ROS	Antioxidant	[102]
		Rats with diabetes produced by STZ	Decreases TNF-α	Anti-inflammatory	[103]
		Rats with diabetes produced by STZ	Decreases IL-6	Anti-inflammatory	[103]
8	*Crocus sativus*	Swiss albino mice treated with genotoxins	Enhanced antioxidants	Antioxidant	[104]
		Guinea pig isolated heart	Blocks Ca^2+^ channels	Vasorelaxant	[105]
		BeCl_2_-treated Wistar rats	Reduces oxidative stress	Antioxidant	[106]
		BeCl_2_-treated Wistar rats	Increases antioxidants	Antioxidant	[106]
		Rats with ischemia–reperfusion	Activates eNOS	Vasorelaxant	[107]
		Rats with ischemia–reperfusion	Inhibits NF-κB	Anti-inflammatory	[107]
9	*Hibiscus sabdariffa*	Healthy men	Lowers uric acid concentration		[108]
		SHR isolated aorta	Increases NO	Vasorelaxant	[23]
		SHR isolated aorta	Blocks Ca^2+^ channels	Vasorelaxant	[23]
		Hypertensive humans in stage 1 and 2	Reduces plasma Na^+^ levels		[30]
		Rat liver with hepatotoxicity induced by CCl^4^	Scavenges ROS	Antioxidant	[109]
		Healthy humans	Enhances antioxidants	Antioxidant	[110]
		Not clear	Increases NO	Vasorelaxant	[111]
		PDGF-treated rat VSMCs	Inhibits ERK pathway activation	Anti-proliferative	[112]
		SHR thoracic aortic VSMCs	Diminished pRb, CDK4 and cyclin D1	Anti-proliferative	[113]
		SHR thoracic aortic VSMCs	Decreases β-galactosidase	Anti-proliferative	[113]
		SHR	Lowers uric acid concentration		[111]
10	*Panax*	Hypoxia/reoxygenation-oxidative injured cardiomyocytes of rat	Increases antioxidants	Antioxidant	[114]
		Mouse cardiomyocytes	Reduces NF-κB	Anti-inflammatory	[115]
		Mouse macrophages	Reduces NF-κB	Anti-inflammatory	[116]
		Mouse macrophages	Decreases TNF-α	Anti-inflammatory	[116]
		Mouse macrophages	Decreases IL-6	Anti-inflammatory	[116]
11	*Salviae miltiorrhizae*	CHD patients	Increases antioxidants	Antioxidant	[117]
		Thoracic aortic VSMCs of Sprague–Dawley rat	Reduces ROS	Antioxidant	[118]
		Endothelial cells of Human umbilical vein	Decreases TNF-α	Anti-inflammatory	[118]
		Endothelial cells of Human umbilical vein	Inhibits NF-κB	Anti-inflammatory	[118]
		Endothelial cells of Human umbilical vein	Inhibits VCAM-1	Anti-inflammatory	[118]
		Sprague–Dawley rat thoracic aortic VSMCs	Inhibits PDGF proliferation	Anti-proliferative	[118]
12	*Zingiber officinale*	Enzymatic assay	Scavenges ROS	Antioxidant	[119]
		Rat heart	Inhibits lipid peroxidation	Antioxidant	[120]
13	*Bidens pilosa L*	High-fructose fed Wistar rats	Mechanism not determined	Vasorelaxant	[64]
		LPS-stimulated RAW 264.7	Inhibits NF-κB	Anti-inflammatory	[121]
		LPS-stimulated RAW 264.7	TNF-α activation	Anti-inflammatory	[122]
14	Mammea africana	l-NAME-induced hypertensive rats	Ca^2+^ antagonists	Vasorelaxant	[123]
15	Cymbopogon citratus	Rat isolated thoracic aorta	Inhibits Ca^2+^ influx	Vasorelaxant	[124]
		Rat isolated thoracic aorta	Increases NO bioavailability	Vasorelaxant	[124]
		Isolated aorta from WKR	Increases NO bioavailability	Vasorelaxant	[125]
		SHR isolated aorta	Increases NO bioavailability	Vasorelaxant	[125]
16	*Nigella sativa*	SHR isolated aorta	Enhances K^+^, Na^+^ and Cl^−^ in urine		[126]
17	*Agastache Mexicana*	Rat thoracic aorta	NO overproduction	Vasorelaxant	[127]
18	Cocos nucifera	Salt-induced hypertensive male Wistar rats	nitric oxide production	Vasorelaxant and antihypertensive	[128]
19	Lepidium sativum	WKY and SHR rats	Na + excretion increased in urine	Antihypertensive and diuretic	[129]
20	Laelia autumnalis	Rat aortic rings isolated	Ca2 + Channels blockade	Vasorelaxant	[130]
21	Carum copticum	Preparations rabbit aorta and jejunum, rat thoracic aorta	Calcium antagonism	Antihypertensive	[131]
22	Olea europaea	Dahl salt-sensitive rat	Angiotensin II inhibition	Antihypertensive	[132]
23	Hsian-tsao	Male SHRs	Increased antioxidant activities	Antihypertensive	[133]
24	Eucommia ulmoides	Dog carotid and rat aortic rings	Nitric oxide production	Vasorelaxant	[134]
25	Phyllanthus urinaria	Spontaneously hypertensive rats	ACE Inhibitors	Antihypertensive	[135]
26	Tropaeolum majus	SHR	Angiotensin II inhibition	Antihypertensive	[136]
27	Fritillaria Ussuriensis	Rat aortic rings	ACE inhibition, increased NO/cGMP level	Antihypertensive	[137]
28	Laelia anceps	SHR aortic rings	Ca^2+^ channels blockade	Vasorelaxant and antihypertensive	[138]
29	Guazuma ulmifolia	Sugar nourished hypertensive rats	Nitric oxide production	Antihypertensive	[139]
30	Lepechinia caulescens	Rat thoracic aorta	Nitric oxide liberation	Vasodilator	[140]
31	Elettaria cardamomum	Preparations of rabbit jejunum	Ca^++^ antagonism	BP lowering effect	[141]
32	Aronia mitchurinii	SHR	ACE inhibition	BP lowering effect	[142]
33	*Momordica charantia*	Rats	Mediate NO/cGMP production	Hypotensive	[143]
34	Clerodendron trichotomum	Rat plasma	ACE inhibiter	Antihypertensive	[144]
35	Tanacetum vulgare	Wistar rat aorta	NO production enhancer	Vasorelaxing	[145]
36	Cecropia pachystachya	Rats hearts	Na,K-ATPase pump stimulator	Cardiotonic	[146]
37	*Eugenia uniflora*	Rats hearts	Ca^++^ antagonism	Hypotensive	[147]
38	*Geum japonicum*	Rat thoracic aorta	Mediate NO/cGMP production	hypotensive	[148]
39	*Cirsium japonicum*	Rat thoracic aorta	Nitric oxide production enhancer	Vasorelaxation	[149]
40	*Astragalus complanatus*	Hypertensive rats	Ang II receptor blocker	Antihypertensive	[150]
41	*Citrus limetta*	Mice	Ang II receptor blocker	BP lowering effect	[151]
42	*Achillea millefolium*	Anesthetized rats	ACE inhibitior	Antihypertensive	[152]
43	Averrhoa carambola	Rat aorta	Ca^2+^ inhibitior	Hypotensive	[153]
44	*Valeriana wallichii*	Preparations of rabbit jejunum	k^+^ channel activation	BP lowering effects	[154]
45	*Erythroxylum gonocladum*	Rat plasma	ACE inhibitior	Antihypertensive	[155]
46	*Cudrania tricuspidata*	Rats with NO synthesis inhibition	NO/cGMP overproduction	Antihypertensive	[137]
47	Antrodia camphorata	Rat aortic rings	NO/cGMP overproduction	Antihypertensive	[156]
48	*Melothria maderaspatana*	Hypertensive rats	Increased vitamin C utilization	Antihypertensive	[157]
49	*Solanum torvum*	Rat aorta	Calcium influx blocked	Antihypertensive	[158]
50	*Echinodorus grandiflorus*	Perfused kidney and aorta of rabbit	Nitric oxide production	Vasodilator	[159]
51	*Polyalthia longifolia*	Rats fed egg yolk	ACE inhibition	Reduced BP	[160]
52	*Jatropha gossypiifolia*	Rat aorta	Ca^2+^/NE antagonism	Antihypertensive	[161]
53	*Salvia cinnabarina*	Male Wistar rats	Nitric oxide production	Lower BP	[162]

**Table 2:** Completed clinical trials of different medicinal plant as anti-hypertensive agents

S. No	Herb	Dose/duration	Condition	Design/population size	Result/magnitude of result	References
1	*Allium sativum*	2600 mg/day garlic powder/10 days	Mild hypertension	Placebo-controlled, crossover/6	SBP diminish/17 mmHg	[38]
		960 mg/day AGE/12 weeks	Uncontrolled hypertension	Double-blind, randomized, parallel, placebo-controlled/50	SBP diminish/10.2 ± 4.3 mmHg	[172]
		480 mg/day AGE/ 12 weeks	Uncontrolled hypertension	Double-blind, randomized, parallel, placebo-controlled/79	SBP diminish/11.8 ± 5.4	[173]
		300–1500 mg/day powdered garlic/4 weeks	Stage 1 hypertension	Randomized, parallel, placebo-controlled/210	SBP and DBP diminish/9.2 and 6.26 mmHg	[174]
2	*Camellia sinensis*	7.6 g tea leaves boiled in 400 ml water/1 h	Mild hypertension	Double-blind, placebo-controlled/20	SBP and DBP enhance/1.7 and 0.9 mmHg (green tea) 0.7 mmHg each (black tea)	[175]
		379 mg green tea extort/ 12 weeks	Obese, hypertension	Randomized, parallel, placebo-controlled/56	SBP and DBP diminish/4 each mmHg	[89]
		4479 mg (3 cups/day, 1493 mg each) black tea/ 24 weeks	Mild hypertension	Randomized, parallel, placebo-controlled/95	SBP and DBP decrease/2 and 2.1 mmHg	[176]
3	*Crataegus* spp.	500 mg/day extort/ 10 weeks	Mild hypertension	Double-blind, randomized, parallel, placebo-controlled/36	DBP/13.1 mmHg	[170]
		2.7–3 mg/day flavonoids (Hydro-alcoholic extract)/4 months	Mild hypertension	Double-blind, randomized, parallel, placebo-controlled/92	SBP and DBP diminish/13 & 8 mmHg	[177]
4	*Crocus sativus*	400 mg/day/7 days	Healthy	Double-blind, randomized, parallel, placebo-controlled/30	MAP & SBP diminish/11 & 5 mmHg	[178]
5	*Hibiscus sabdariffa*	10 g/day dehydrated calyx/4 weeks	Mild to moderate hypertension	Randomized, captopril-controlled 75	SBP & DBP diminish/15.32 & 11.29 mmHg	[29]
		720 mL/day (3 times, 240 mL each) tea form/6 weeks	Pre- and mild hypertension	Randomized, double-blind, placebo-controlled/65	MAP, SBP, & DBP diminish/7.2, 3.1, & 4.5 mmHg	[31]
6	*Nigella sativa*	200 & 400 mg/day seed extract aqueous (100 and 200 twofold a day)/8 weeks	Mild hypertension	Randomized, double-blind, placebo-controlled/108	SBP & DBP diminish/2.2 & 1.1 mmHg LDL-cholestrol reduction	[179]
		5 mL/day (2.5 double a day) *Nigella sativa* oil/8 weeks	Healthy	Double-blind, randomized, parallel, placebo-controlled/70	SBP & DBP diminish/ 10.6 & 9.6 mmHg	[180]
7	*Panax*	3 g/day *P. quinquefolius/*12 weeks	Essential hypertension	Randomized, double-blind, placebo-controlled/64	SBP decrease/17.4 mmHg	[181]
		300 mg/day *P. ginseng* extract/8 weeks	Mild hypertension	Randomized, placebo-controlled/ 90	SBP and DBP decrease/3.1 and 2.3 mmHg	[182]
		400 mg/ 3 h	Healthy	Randomized, double-blind, crossover/23	SBP and DBP decrease/4.8 and 3.6 mmHg	[183]

#### ***Hibiscus Sabdariffa*** (HS) (Family: Malvacae; Common Name: Rosella, Hibiscus, Jamaica Sorrel, Red Sorrel) [16–19]

The various part of this plant like flower, leaves and calyxareare used for thetreatment of various medicinal problems in manyWest African countries [20]. Due to its pleasing taste, decorative appearance, medicinal and culinary effect, HS is used worldwide to produce many types of modern cold and hot drinks. Tender young leaves, calyx and stems are used as salads in raw or cooked form. At many places, calyx is used to prepare soups, pickles, sauces, pudding and also as flavoring agents. The Nigerian citizens used calyx infusion (zobo) as an antihypertensive agent.

Experimental studies showed that HS has antimicrobial, antioxidant, anticholesterol, antihypertensive activity [21]. The people of Jordan’s North Badialocality use leaves and flowers of HS, and according to them, hot aqua infusions are used to treat elevated blood pressure while cold infusions for lower blood pressure [22]. In Tobago and Trinidad area’s resident’s leaves are used to treat hypertension while the flower and seeds are used forhypocholesterolemic effect [23, 24].

Previous studies showed that on treatment with HS the SBP and DBP level declined dose dependently in salt induced hypertensive and in the normotensive group [25]. When comparing to ACE-inhibitors, it was equally effective to captopril [26] but less effective than lisinopril [27].

From different studies, it is found that extract of calyces of HS has antihypertensive and vasodilator effect in human and experimental animals*via*vasodilator pathways dependent and independent on theendothelium. The opening of thecGMP/nitric oxide-relaxant pathwayis derived via endothelium causesendothelium-dependent vasodilation by activation of guanylate cyclase whereas inhibition of Ca^2+^ influx is responsible for endothelium independentcomponent [20].

The watersoluble active constituents of HS, anthocyanins, predominantlycyanidin-3-sambubiosideand delphinidin-3-sambubioside, are responsible for the hypocholesterolemic, antioxidant & antihypertensive effects [24, 26–29].
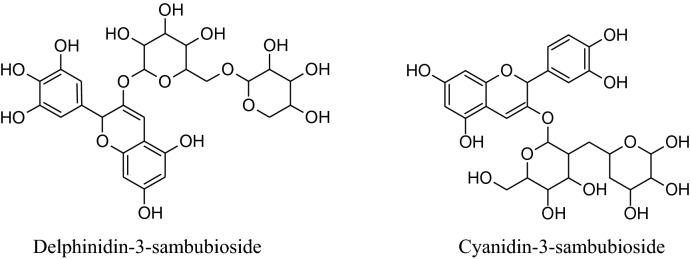


#### *Allium Sativum*: (Common Name: Garlic; Family: Alliaceae or Liliaceae) [30, 31]

The bulb of *A. Sativum is* a multipurpose spice or herbpopularly used for thousands of yearsas a vegetable because of its strong flavor and taste worldwide. It is an herb of interest for the treatment of cardiovascular diseases (CVDs) like coronary heart disease, hypertension, atherosclerosis and age-related vascular changes [32]. It can also use as an antioxidant, anti-cancer, anti-inflammatory, antibacterial, and hypocholesterolemic. All these pharmacological activities make it interesting for pharmacologists and health practitioners.

The presence of organosulfur constituents like allicin (major active constituent), ajoene, S-allyl-l-cysteine, diallyl disulfides (DADS), methyl thiosulfonate and diallyl trisulfides etc. are responsible for these pharmacological activities.*Allium Sativum* can be utilized in diversevarietiessuch as raw,dried powder, aqueous extract, oil and aged garlic extract(AGE) form. Mata analysis interpretation confirmed that AGE produces a dependable lowering of blood pressure (both SBP and DBP) compared to other forms of *A. Sativum* [33].
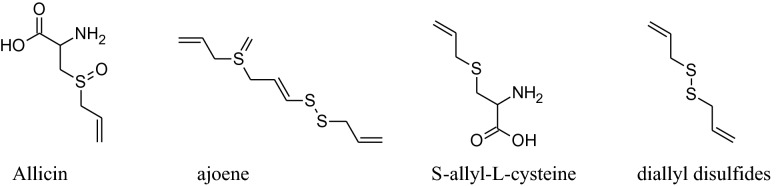



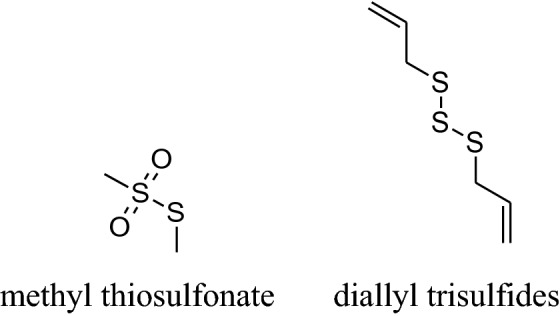


Ku et al. and Mousa and Mousa reported that garlic’s ethanolic extract caused relaxationby sulfide components like allicin in pulmonary arteries of rat *via*increasing the NO bioavailability [34, 35]. Benavides et al. reported that garlic provides polysulfides to red blood cells to boost H_2_S production and synthesis results vasorelaxation [36].

Moreover, Sendlet al. reported that garlic’s component gamma-glutamyl-cysteines acts asan antagonist to inhibit ACE activity [37]. When Alliin reacts with the Alliinase enzyme, Allicin antagonizesthe endothelin-1 effect, decreases vasoconstrictor responses of Ang II anddeactivate NF-kB [33, 38–42].

#### ***Andrographispaniculata***: (Common Name: Kalmegh, Kirayat, Bhunimba, King of Bitter; Family: Acanthaceae)

This is a traditional medicinal plant of eastern and southeastern Asia commonly used for treating cold, fever [43], upper respiratory and gastrointestinal tract infections, hepatitis, herpes and CVDs [44].

*Andrographis paniculate* actsby inhibitingthe activity of β- adrenoceptors, autonomic ganglion receptor and angiotensin converting enzyme (ACE) [45]. Its extracts contain several diterpenoid compounds *i.e*. 14-deoxy- 11,12-didehydroandrographolide, andrographolideand14-deoxyandrographolide [46] responsible for anti-inflammatory,bactericidal [47], antioxidant and hypotensive effects. Its chloroform extract can activate NO synthesis and finally stimulate NO production in endothelial cells which ultimately cause relaxation in smooth muscles by inhibiting Ach action [48]. *A. paniculata*decrease BP by decreasing reactive oxygen species and ACE activities in impulsively hypertensive rats (SHR) [44]. According to Awang et al. [46] found that vascular resistance reduced in isolated rat heart by both 14- deoxyandrographolide and 14-deoxy-11,12-didehydroandrographolide. According to them, crude extract containing a high concentration of 14-deoxy-11,12-didehydroandrographolide produce remarkable hypotensive property*via* increased NO release which is responsible for vasodilation. Moreover, 14-deoxy-11,12- didehydroandrographolide decrease the level of Ca^2+^ inside cell by voltage-gated Ca^2+^ channels.The chloroform extract of *Andrographis paniculata* blocks the L-type Ca^2+^ current and high K^+^ activation pathways produced endothelial protective effects to relax the smooth muscle and the results were comparable to verapamil [33, 49–51].
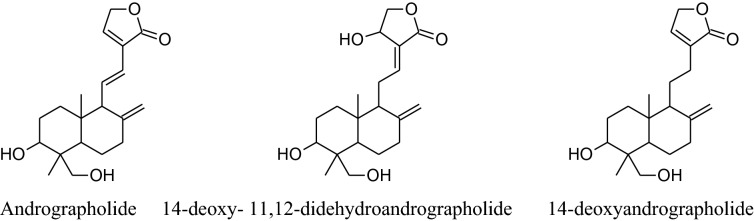


#### *Apiumgraveolens* (Celery): (Common Name: Celery, Ajmod; Family: Umbelliferaeor Apiaceae)

It is anyearly or permanent herbs commonly found in sub-tropical and temperate parts of Asia and Africa. Different parts of*Apium graveolens* are used for the preparation different medicinal formulations because of its anti-inflammatory, anti-hypertensive, anti-microbial, bactericidal, fungicidal, anti-cancer, anti-virus, gastro-intestinal, anti-spasmodic and anti-oxidant properties [52].

In vivo animal studies showed that *A. graveolens* hasa hypotensive effect [33]. According to Moghadam et al. its hexaneextracts of seed decrease blood pressure more effectively in hypertensive rats than its additional extracts [53]. This is because of betterpreservation of n-butylphthalide, which is responsible for the flavor and aromatic odor of celery. This effect of n-butylphthalide was also supported by SHRs [54]. According to Ko et al. apigeninflavoneextracted form effect voltage and receptor gated channels via blocking of Ca^2+^ influxwhich in result block aortic ring contractions in the isolated aorta of rat [55]. Houston reported that active components of celery lower human arterial pressure probably by the declining the intensity of circulating catecholamines and lowering vascular resistance [56]. Fazal et al. reported that daily use of seed extract for 4 weeks can reduce blood pressure by 12% [57]. Moreover, Popovic et al*.* reported that the flavonoid content of this herb reduces oxidative stress which can potentiate antioxidant mechanisms [33, 58]. Moghadam et al. confirmed that celery seeds have a hypotensive effect due to some hydrophobic components, such as n-butylphthalide (NBP) [50, 53]. It can also use to treat hypertension associated with the liver [51].
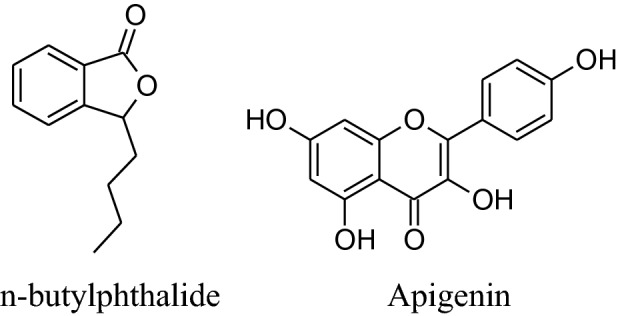


#### *Bidenspilosa L*. (Common Names: Broom Stick, Beggar’stick and Black-Jack; Family Asteraceae)

The whole plant of *B. pilosa*plant are used as components in folk medicines in different form as a tincture, dry powder, maceration or decoction.

The bioactive components have many health benefits andused inbacterial, cancer, obesity, hypertension, malarial and cardiovascular diseases [12, 59] which make it plant of interest nowadays. Different parts of *Bidens pilosa* contain numerous useful chemical constituents with at least 60 flavonoids [12]. So, normally extracts of this plant are used as medicine to treat around 40 categories of illnesses [12, 60] by different expected [2] mechanism like vasodilatation, lipid profileimproving, free radicals scavenging insulin-sensibility,calcium blocker etc.[61–64]. Previous studies have confirmed that quercetin increases the NO production and/or bioavailability which improved endothelium function. Additionally, Bilanda et al. supported it that quercetin can attenuate and prevent hypertension [2]. *Bidens pilosa’s* methylene chloride and aqueous extracts inverted the hypertriglyceridemia and high blood pressure produced by fructose feeding but does not affect plasma levels of glucose and insulin but a fewexperiments showing effect on insulin sensitivity [12, 61]. According to Gulfsha et al. high doses of leaf extracts of *Bidens pilosa*can decreaselevels of plasma creatinine which in result increases the level of plasma cholesterol. So, they suggested that *Bidens pilosa’s* hypotensive effect is independent of insulin sensitivity [65].

According to Dimo et al. aqueous and CH_3_Cl leaves extort of *Bidens pilosa can* attenuate and averthigh blood pressure in various normotensive and hypertensive rat models (induced by fructose) for a 3-week continuous treatment [2, 66]. Dimo et al. and Bartolome et al. proved that *B. pilosa has* vasorelaxant responses [12, 61] and it is also supported by Nguelefack et al. as they reported mounting amounts of a neutral to extort of *B. pilosa*causerelaxation in noradrenaline and potassium chloride pre-constricted aortas of a rat. But they did not interpret a clear mechanism to explain vasodilation [67]. Their assumptions were vasodilation possibly happen either by calcium channel antagonism or involve cyclooxygenase metabolite [33]. They also mentioned that mechanism of vasodilation was not associated with the ATP-dependent K^+^ channel [12, 67].
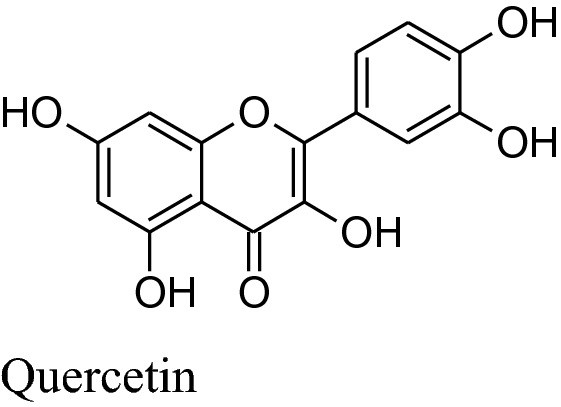


#### *Camellia sinensis* (Common Name: Tea; Family: Theaceae)

New clean and bright leaves of *Camellia assamica*or *Camellia sinensis*arecharacteristically processed to prepare the most frequently consumed beverages *i.e.* tea, worldwide which is next to the water.

The major flavonoids of tea are catechins which include (−)-epicatechin-3-gallate (ECG), (−)-epigallocatechin-3-gallate (EGCG), (−)- epicatechin (EC), (−)- epigallocatechin (EGC, primary component) [68]. These catechins are converted to flavins and the arubigins, by an enzymatic reaction, are known as effective vasodilators. These catechins are also responsible for major elevation in blood flow by increased liberation of NO through a simultaneous decline in intensities of oxidative stress and dimethylarginine [69]. Moreover, Hong et al. reported that EGCG was capable of reduceactivation of an NF- kB in endothelial cells of humans. It has the secondary metabolites like purine alkaloids, phenolic acids, flavan-3-ols, flavonols, saponins, hydrolyzable tannins, and condensed tannins as well as their glycoside forms. Many chemical constituents such as flavan-3-ols derivatives, theaflavins, thearubigins, etc*.* are emerged to form new constituents with a change in the concentration of others.Sodepending on the process each type of tea has different flavorsand constituents. Aqueous extract of *Camellia sinensis* can produce pleiotropic effects as well as anti-diabetic, anti-inflammatory antibacterial, antihypertensive and anti-cancer activities [70]. Deka and Vita, according to them the person consuming regularly green and black tea having a minor risk of hypertension [68]. Peng et al. also reported based on meta-analysis that regular use of tea has a major decreasing result on diastolic blood pressure (DBP) [71], other studies showed that it is concentration dependent [72]. According toa Japanese group analysis, regular consumption of concentrated, only green tea can reduce the CVD mortality risk [73]. The *o*-methylated EGCG content of tea can inhibit angiotensin-converting enzyme therefore consumption of black tea extract by regular 7 days has a decreasing effect on systolic blood pressure (SBP). Cheang et al. on basis of their study explain that theaflavin-3,3-digallate can inhibit acetylcholine dependent contraction and enhance endothelial functionby reducingthe stress of endoplasmic reticulum and modifyingHcyenzymes such as cystathionine gamma-lyase and cystathionine-*β*-synthase. By consumption of lyophilized extract of green tea, a majordecline in SBP (− 4.9 mmHg) andDBP (− 4.7 mmHg) in slightly hypertensive patients [74, 75]. According to Peng et al. gree tea can reduce blood pressure by different mechanisms such as by maintaining balance among vasoconstricting, vasodilating and hyperpolarizing factors [71]. It has a rising effect on the production of nitric oxide (NO) to enhance ventricular function and manage ROS production by provoking antioxidant enzymes and reducing pro-oxidant enzymes [76].


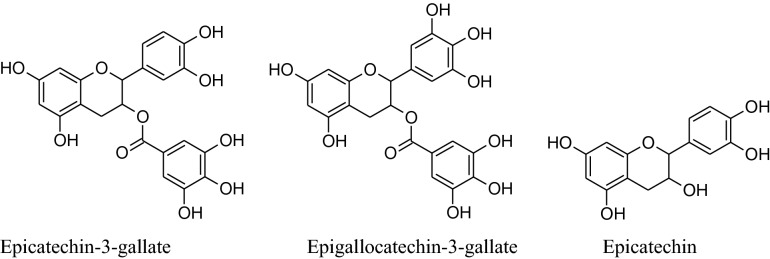


#### *Coptis chinensis* (Family: Ranunculaceae, Common Name: Chinese Gold Thread)

It is utilized in Chinese folk medicine. Main chemical constituent of *Coptis chinensis is* berberine which is responsible for its major pharmacological activities such as sedative, immunostimulatory, hypotensive, antimicrobial, choleretic, anticonvulsant, uterotonic, antihelminthic, anticancer and carminative activities. Moreover, it also affects lipid and carbohydrate metabolism, cardiotonicity and endothelial function. Because of all these activities, in the last decade, this alkaloid is a point of interest for researchers [76].

**Fig. 7:**
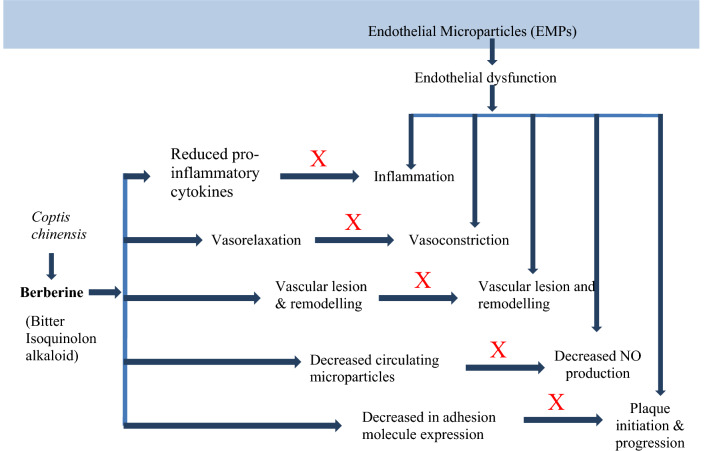
Mechanism of action of berberine


Lan et al. confirmed that berberine has a major hypotensive effect by numerous mechanisms. BBR raises the expression of enzyme eNOS which is associated with synthesis and release of NO followed by vasodilation. This vasodilation is possibly arbitrated by well known vasodilator PGI2 and KATP channels opening as well as Ca^2+^ influx blockage. One study reported thatBBReffects endothelial dysfunction by reducing the maturity of endothelial microparticles. Berberine can also inhibit transcription factor NF-kB and VCAM-1expression as well as VSMC proliferation [163] (Fig. 7).

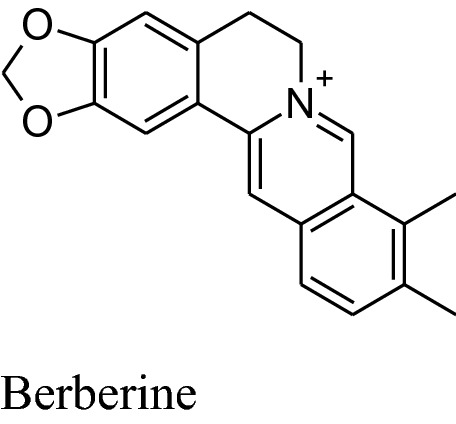


#### *Coriandrum sativum* (Family: Umbelliferae/Apiaceae; Common Name: Kasbour, Coriander, Cilantro) [164]

The fruits and leaves of *C. sativum* and *C. tordylium* are used in the conventional treatment of different gastrointestinal and heart diseases and as well as catering ingredient.Its oil is used in many cosmetics formulation. Usually, *C. sativum* used for the treatment of many GIT disorders likeflatulence, diarrhea, anorexia, dyspepsia, vomiting and pain as well as an antihypertensive, antiseptic,antiemetic, myorelaxant, antidiabetic, anti-inflammatory, emmenagogue, lipolytic and possess nervesoothing effect.

The major chemical constituents of coriander are linalool, geranyl acetate and gamma-terpinene. It also have other chemical constituents like a-cedrene (3.87%), citronellal (1.96%), geraniol (1.87%), b-pinene (1.82%), b-sesquiphell-andrene (1.56%), citral and Citronellyl acetate (1.36% each), citronellol (1.31%), m-cymene (1.27%) and a-farnesence (1.22%) as minor. Till date coriander havebeen not tested in clinical trials to evaluate its result on BP but, it is reported in many studies that coriander shows antioxidant activities and inhibits ROS production by b-adrenoceptor. Jabeen et al. reported that vasodilatory effects of dilute methanolic extort of well dried seeds and powder of coriander produced and mean arterial blood pressure, SBP and DBP fall in a dose-dependent manner in normotensive Sprague–Dawley rats [165]. The vasodilator effect occurs through Ca^++^ channel blockade and endothelial-dependent pathways [166]. The active constituents act synergistically to balance vasoactive constituent for management and treatmentof hypertension.Also, coriander extract has an inhibitory effect on NF-kB and Inos [166].
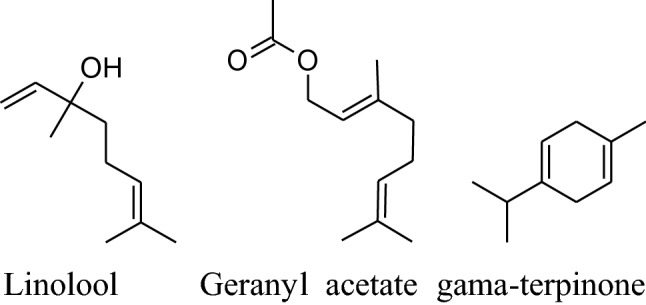


#### *Crataegus *spp. (Genus: *Crataeguscrenulata*Syn., *Pyracanthacrenulata*; Family: Rose; Common Name: Hawthorns, Hawberry or Thorn Apple)

Hawthorns shrubs areemployed in conventional medicine for long years for the handling of CVDs. Walker et al. reported that hawthorns drug (500 mg for regular 10 weeks) can decreases in DBP in hypertensive patients [167]. According to Bone and Mills major reduction in BP occurs only after administration of drug in higher doses for longer duration of time [168].

Asgary et al. run a random, placebo-controlled, double-blind clinical trial by the administration of *Crataegus curvisepala’s*hydro-alcoholic extracts of flowers for three months found that both DBP and SBP decreased by around 8 and 13 mmHg, respectively. The major chemical constituent of *Crataegustanacetifolia*isquercetin, a polyphenolic flavonoid, which is responsible for its major pharmacological functions a vasorelaxant, anti-inflammatory and anti oxidanteffects.Other multiple components of these plants are oligomeric proanthocyanidins*i.e.* procyanid in, procyanidin B-2, hyperoside etc. and flavonoids *i.e.* vitexin,rutin, etc. Moreover, extracts of hawthorn iseffectual on both endothelial cells and VSMCs. *Crataegustanacetifolia’s*extract cause vasodilation by increasing phosphorylation and activation of eNOS at serine1177 which in result enhances synthesis and release of NO in endothelial cells [169]. Anselm et al*.* informed that flower and leaves extract and segregate, hyperosideof Crataegus, probably, can activate multiple signaling pathways, as PI3-kinase,eNOS, Src, ROS, Akt and up-regulation of antioxidant enzymes (CAT, SOD) to produce endothelium-dependent relaxation.Together with the contribution of all of the mechanisms of actions ameliorate *Crataegus’s* hypertensive outcome. Interestingly, the extract has anti-inflammatory action also by decreasing the level ofVCAM-1, IL-6, NF-kB, iNOS andTNF-a [170, 171].


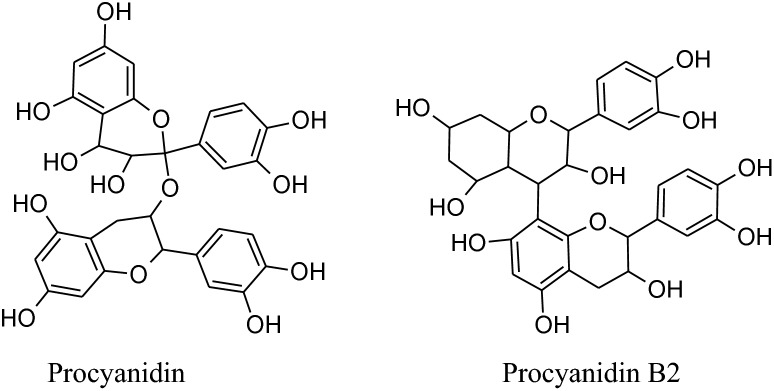



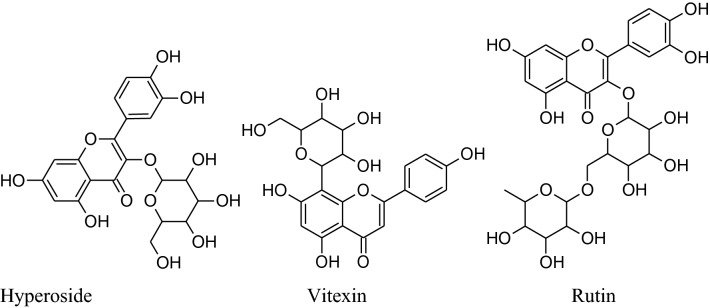


#### *Crocus sativus* (Family: Iridaceae; Common Name: Saffron)

*Crocus sativus* isanherb without stem has medicinal values for over 4000 years. It is used for pleasant flavor and color in different foods and in cosmetology also. It has chemical constituents such as flavonols (kaempferol) carotenoids (crocin and crocetin), phenolic compounds, anthocyanins, terpenoids and alkaloids. Commonly, the extract of saffron was used as an antispasmodic, aphrodisiac, expectorant, anti-depressant, antitussive, anticonvulsant, neuroprotective, hypolipidemic, anxiolytic, anticancer, cardiovascular protective and antioxidant [177].

Its main chemical constituents are crocin, safranal, picrocrocin and crocetin. These components act as anti-hypertensive by different mechanisms of action. Modaghegh et al. reported that regular use of saffron for 7 days can cause a major fall in arterial pressure and SBP in healthy humans because of its vasorelaxant action [184]. According to Fatehi et al. petal’s extracts of *C. sativus* contain high concentrationof anthocyanins and flavonoids can modulate peripheral vascular resistance which in response reduces BP of male Sprague–Dawley rats in quantity dependent method [185, 186]. Imenshahidi et al. reported that crocin, safranal and stigma extract of *C. sativus* calm down mean blood pressure in normotensive as well as salinestimulated hypertension in male Wistar rats [177]. Later on, in 2015, they also reported that safranal on chronic administration can reduce SBP in salt hypertensive rats but didn’t affect normotensive one.Boskabady et al. mentioned that chemical constituents of saffron mainly crocinreduced contraction and heart rate of guinea-pig*via* potassium channels opening, Ca^2+^ channels blocking and b-adrenoceptors antagonism. Moreover, safranal also affect protein kinase B phosphorylation/ GSK-3b, activation of iNOS, TNF-a expression and NF- kB activity [4, 178].
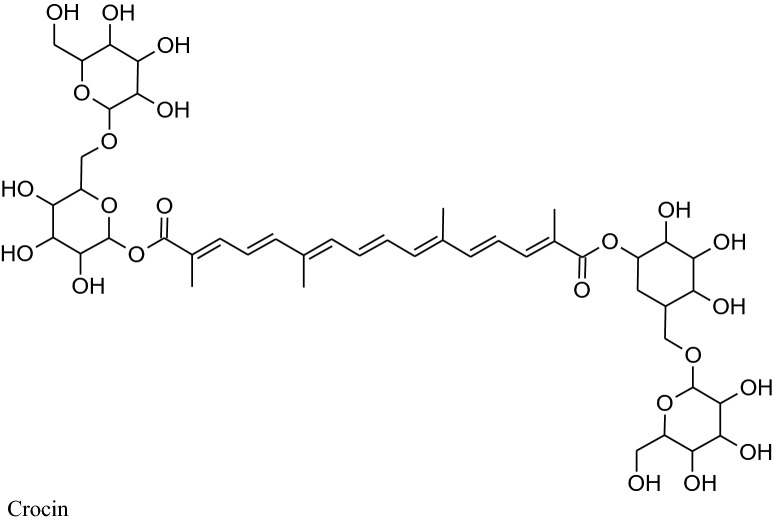



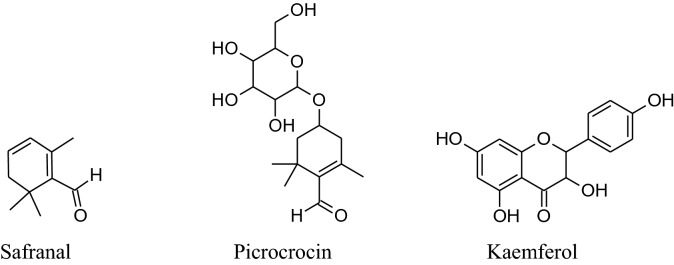


#### *Cymbopogon citrates* (Family: Gramineae; Common Name: Lemongrass, Citronella, Squinant)

Extract of shoot and leaves of *C. citrates* has been widely used for its nutritional, cosmetic and medicinal applications, globally for the high contentof essential oil. Various studies acknowledged the occurrence of its phytoconstituents such as flavonoids, alkaloids, essential oil, phenols, tannins, deoxysugars, saponins, anthraquinones in the leaves and stem of herb [187].

The major constituent of *C. citrates* is citral which is alone or in combination with other componentshas been used as antimicrobial, antioxidant, chemo-protective and antispasmodic properties.Chitra Devi et al*.* reported that methanolic extracts of aerial parts (stems & leaves) and roots *C. citrates* (having citral as the main active constituent) displayed vasorelaxation on the phenylephrine-stimulated contractions in a dose-dependent manner. Citral effects synthesis and release of NO to produce vasorelaxantion by inhibiting the attenuation caused by L-NAME. Moreover, leaves extract may affectsthe synthesis of prostacyclin to induce relaxation. Furthermore, the relaxant effect of the combination of the extract of root, stems and leaves may be due to the blockage of Ca^2+^ ion channels (endothelium-independent) [188]. Fresh leaf extract of Cymbopogon citrates in combination with other herbal medicines like fruits’extract of Citrus medica and fresh leaf extract of Persea americanacan reduce hypertension in rats induced by sucrose and ethanol. This mixture can be used to protect kidney, liver and vascular endothelium damaged by chronic utilization of sucroseand ethanol [105]. Ray reported that decoction of lemongrass has been produced a major effect on mean arterial pressure on the administration of twice-a-day [189]. From the dairy of Bastos et al. an intravenous bolus inoculation of citronellol (acyclic monoterpenoid) formed an antihypertensive effect in Wistar rats by blocking calcium channels as well asmodifying caffeine-gated and IP_3_ dependent intracellular stores of Ca^2+^. Lemongrass oil can capability to suppress the activity of ROS. Interestingly, citral reduces iNOS and NF-kB activity and produce anti-inflammatory actions [125].
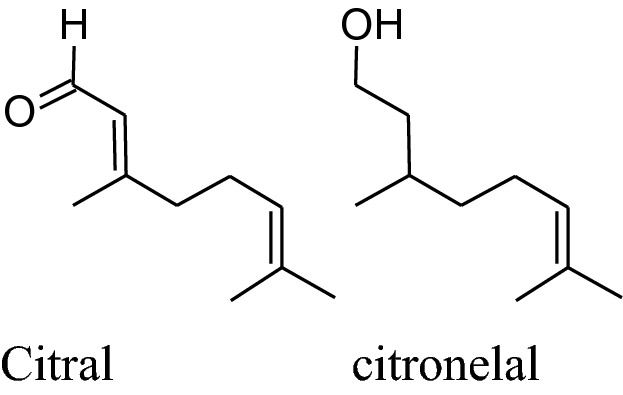


#### *Nigella sativa* (Family: Ranunculaceae; Common Name: Seed of Blessing, Black Cumin)

Different aqueous and oily ofSeed of Blessingdisplay a broad range of pharmacological activities and used to treat many ailments and disorders like diabetes, inflammation, hypertension, cardiovascular complications, hepatic disorder, cancer, kidney disorder and arthritis. *N. sativa* has a decreasing effect on blood pressure [7, 190]. The essential oil of black seed has thymoquinone, as a major active constituent liable to most of the valuable effects of seeds [124, 190, 191]. According to Jaarin et al*.* Black Cumin’soil has antihypertensive effect by reducing ACE in vivo [192]. Thymol, The other active component of *N. sativa,* has been reported to decrease blood pressure by endothelial independent pathway (inhibit the influx of calcium ions through calcium channels) in the membrane of endothelium cells followed by vasorelaxation [7].

The black seed oil has four significant, pharmacologically active compounds: thymol (THY), thymoquinone (TQ), thymohydroquinone (THQ), dithymoquinone (DTQ) and α-hederin, essential oils, flavonoids, antioxidants, alkaloids, saponin, proteins, fatty acids*etc*.are other bioactive components. Wong reported that regular use of the extract of *N. sativa* twice daily for eight weeks in mild hypertension results in a significant decrease in blood pressure [193]. *Nigella sativa* has an inhibitory action on reactive oxygen species can play a possible role in the management of hypertension [194]. Huseini et al. concluded that the oil of *N. sativa* can considerably decreases both DBP and SBP [195]. Besides, Ahmad et al*.* explained that TQ cause vasodilation by reducing synthesis and release of metabolites of COX-1 and COX-2 [124]. Black cumin has an inhibiting effect on NF-kB and TNF-a to act as an anti-inflammatory agent [33, 196].
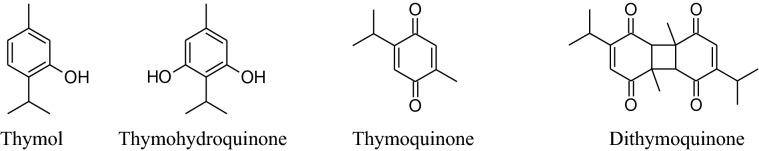


#### *Panax *(*Panax ginseng, Panaxquinquefolius, Panax japonicas, Panaxnotoginseng;* family: Araliaceae; Common Name: Japanese Ginseng, Asian or Korean Ginseng, Chinese ginseng, American Ginseng)

The Panax (“all healing”) has self-confidence traditionally to heal all ill health problems of the human body [197]. Roots of Panaxmainly used in folk medicine for amplevariety of pharmacological and therapeutic purposes for centuries either in solid form or in liquid. Till date, some of 40 ginsenosides have been discovered most active and useful of them are Rb1, Rg1, Rg3, Rh1, Re, and Rd [180]. This medicinal plant has many biological benefits as hypotensive, antioxidation, antidiabetic, vasorelaxation, anti-carcinogenic, anti-allergic, anti-inflammatory, antidiabetic, anticancer, etc.[197, 198]. Amazingly, Kim reported that *ginseng* can “normalize” hypertensive and hypotensive conditions. It also acts as an anti-carcinogenic and antidiabetic agent [180]. This is well reported in the literature that *ginseng* has a reducing effect on blood pressure but according to Mucalo*et al.* it can also increase blood pressure to regularize hypotensive conditions rheostatically probably by alteration of vascular character, adjustingANS, or adapting baroreflex of arteries [199]. Rhee et al*.* found that ginsenoside of *P. ginseng* can cause a major decreasing effectin SBP and DBP in patients having gentle hypertension as well as healthy subjects [200]. Ginsenoside Rg3 produces an increasing effect onexpression of eNOS leads to an increase in the production of NO followed by vasorelaxation [201]. Also, *ginseng* also inhibits adrenal catecholamines emission, which has an additional effect on antihypertensive character [180].
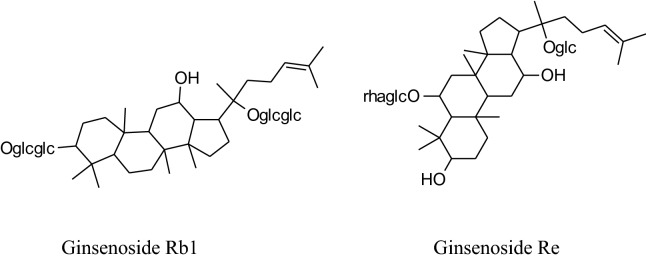



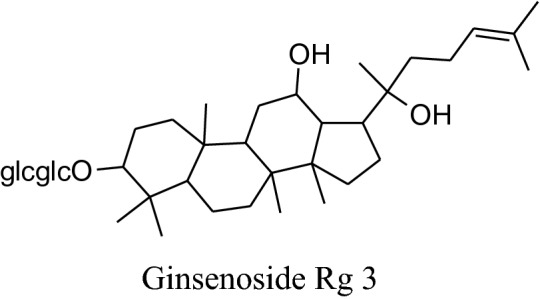


#### *Salviaemiltiorrhizae* (Family: Labiatae; Common Name: Danshen, Red/Chinese Sage)

*Salviaemiltiorrhizae* is oldest and regularly used traditional herbs of China generally utilized for CVDs treatment. Its major phytochemicals are danshensu, tanshinones (tanshinone I &tanshinone II) salvianolic acids (A & B) with other compounds as minor. Mainly root extracts, hold beneficial pharmacological behavior like anti-microbial, antiviral, anti-oxidant, anti-cancer, anti-inflammatory activity and cardiovascular diseases [181, 182]. Danshen’s roots extract diminish pulse rate and systolic blood pressure [202], moderately via enhancing the synthesis of eNOS signaling and amplify NO production to producevasodilation. Tanshinone IIA causes vasodilation without involvement of endothelium cells mechanism [202]. Wang et al. reported that danshen’s metabolite increases stored as well as the influx of Ca^2+^ intracellular*via*a ffecting receptor and voltagedependent calcium channels [203]. Danshen also inhibits ACEs to cause a reduction in blood pressure [149, 204] results in scientific antihypertensive effectsdocumented that danshen also affects other parameters involved in hypertension such as ROS production, oxidation, inflammation and proliferation [205–208].
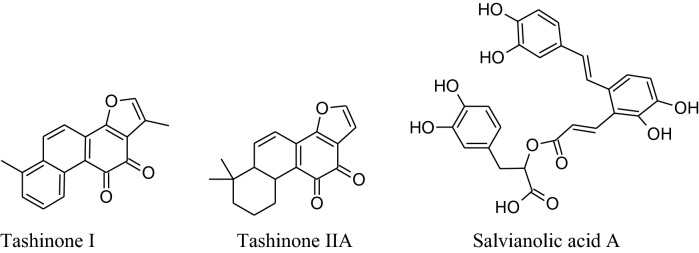



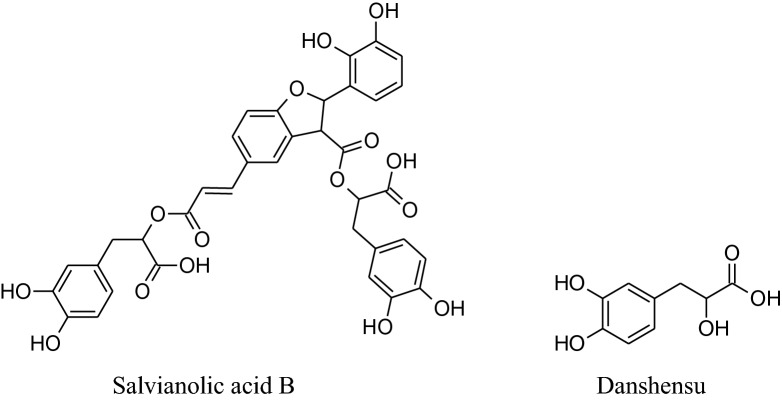


#### *Zingiberofficinale* (Family: Zingiberaceae; Common Name: Ginger)

Rhizome of *Zingiber officinale,* a very commonly used culinary ingredient last for thirteenth century [209]. Akinyemi et al. reported that ginger’s aqueous extract can reduce ACE and lipid peroxidation [210].Suekawa et al. had been found that intravenous and oral dose of (6)-shogoal and (6)-gingerol produced a significant decline in BP [211].

Definitely, [6]-ginger oils are considered to be a fresh antagonist to the angiotensin IItype1 receptor to produce vasodialation [212]. *Zingiber officinale *(*ZO*) has a long history of traditional use. It includes numerous components name as beta-carotene, gingerdiol, gingerol, gingerdione, caffeic acid, capsaicin and curcumin. The literature survey confirmed that ginger has multiple biological activities, counting blood pressure-lowering, antioxidant, cholesterol-lowering, anti-inflammatory, antimicrobial, anticancer, antiplatelet aggregation, hypoglycemic, cardiovascular protective,neuroprotective, respiratory protective, antidiabetic, chemopreventive, antiobesity, antiemetic, antinausea [120]. The health profits of ginger are mainly credited to the presence of phenolic compounds like shogaol and gingerols. Ojulari*et al.,* (2014)concluded that *Zingiber officinale* use can reduce BP [213].Talaei*et al.* showed that daily use of powder of ginger for 56 days can lower DBP and SBP in patients having type 2 diabetes [14]. Some studies proved that ginger can be used with antihypertensive drugs for the treatment of hypertension to provide an addition effect [92].
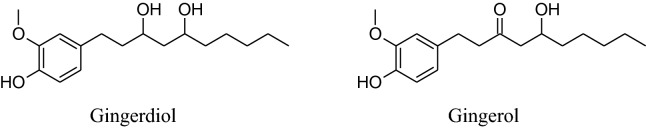



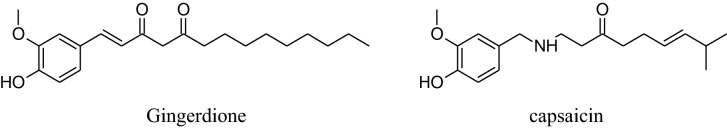



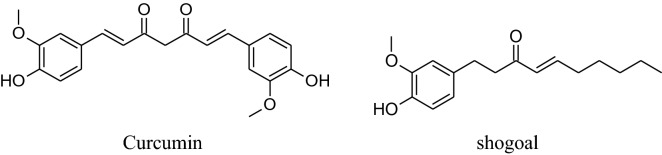


#### *Tribulus terrestris* (Family: Zygophyllaceae; Common Name:Gokhru/Gokshura, Puncture Vine)

The annual shrub, *Tribulusterrestris* has been used as medicine for a long time to treat various types of ailments. Different parts of shrub contain a range of medicinally important chemical constituents which as flavonol, spirostanol and furostanol saponins (tigogenin, neotigogenin, hecogenin, neohecogenin, gitogenin, neogitogenin, chlorogenin, sarsasapogenin, ruscogenin, and diosgenin), flavonoids, alkaloids and glycosides (quercetin 3‑*O*‑rutinoside, quercetin 3‑*O*‑glycoside and kaempferol 3‑*O*‑glycoside) [214]. These active constituents showed immunomodulatory, aphrodisiac, antiurolithic, diuretic, hypolipidemic, antidiabetic, hepatoprotective, analgesic, absorption enhancing, cardiotonic, anti‑inflammatory, antibacterial, antispasmodic, anticancer, anticariogenic, larvicidal and anthelmintic activities [214]. According to Chui et al. and Lu et al*. Tribulus terrestris* herb used habitually for the treatment of coronary heart disease, cerebral arteriosclerosis, myocardial infarction, thrombosis and hypertension [215–217]. Aqueous and methanolic extracts of gokhru possess an imperative antihypertensive effect directly by membrane hyperpolarization and relaxation of arterial smooth muscle in impulsive hypertensive rats [214]. Adaikan*et al*. reported that beneficial effects for the treatment of different ailments are credited to its capability to boost up the discharge of nitric oxide (NO) from the nitrergic nerve endings and endothelium [218]. Also, Sharifi, et al. recommended that the antihypertensive effect of gokhru may be associated with its angiotensin converting enzyme (ACE) inhibitor action [217, 219].
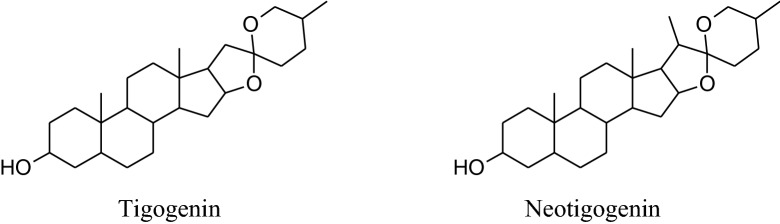



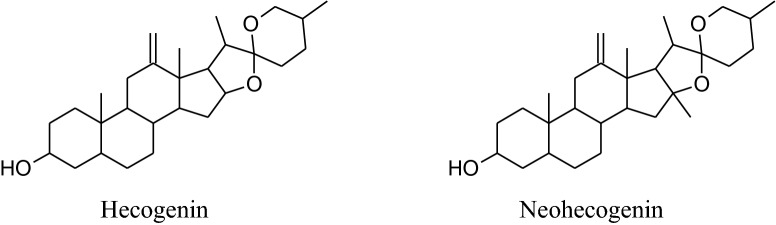



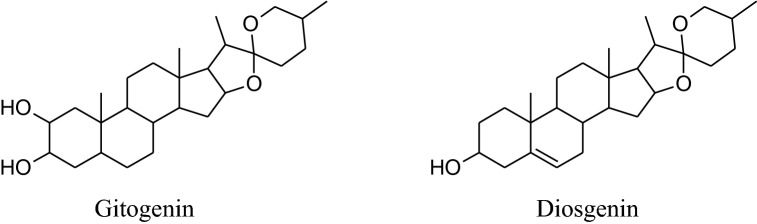


#### *RauwolfiaSerpentina* (Family: Apocynaceae/Dogbane; Common Name: Devil Pepper, Indian Snakeroot, Serpentine Wood)

*RauwolfiaSerpentina* is mainly used to treat hypertension. It slows down the activity ofnervous system which in result decreases heart rate and dilates blood vessels. Rauwolfia consists of indolealkaloids as major phytochemical with others including fatty acids, alcohols, sugars and glycosides, steroids, phytosterols, flavonoids, oleoresins and tannins.indolealkaloids are present in all parts of the plant but major source is root’s bark. The identified different indole derivatives are ajmalinine, ajmalidine, ajmalicine, ajmaline, coryanthine, aricine, deserpidine, canescine, lankanescine, isoserine, isoajmaline, isoserpiline, rauhimbine, neoajmaline, raubasine, papaverine, raucaffricine, reserpine, recanescine, reserpiline, rauwolfinine, rescinnamine, thebaine, serpentinine, reserpinine, serpentine, yohimbine, sarpagine and yohimbinine [220]. In all the above indole derivatives, reserpine is the major one and has antihypertensive activity as can reduce both systolic and diastolic blood pressure [220–223]. Reserpine has the irreversible binding capacity to VMAT2 results in biogenic amines depletion e.g. serotonin, nor-adrenaline and dopamine level in VTA (ventral tegmental area), hypothalamus and nucleus accumbens.Molecular mechanism shows that VMAT2 protein irreversibly binds to storage vesicles in cell and causes ‘leak’ their content, *e.g.*monoamine, into the cytosol which is then tainted by MAO-A enzymes. According to this mechanism renovation of monoamines is independent of age [224].
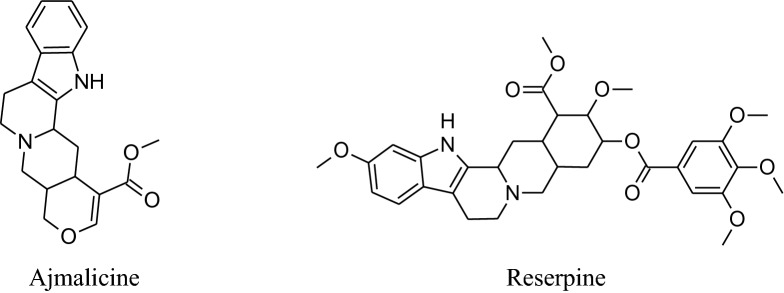



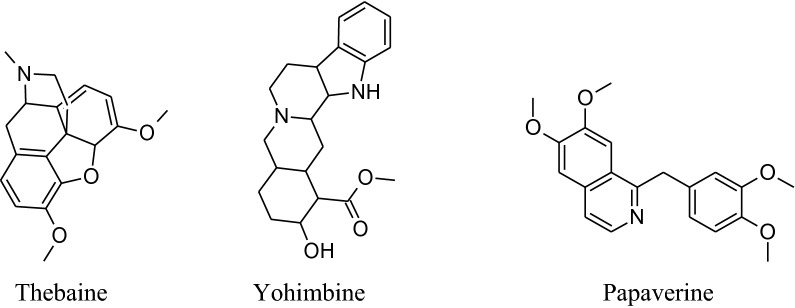


#### *Terminalia arjuna* (Family: Combretaceae; Common Name: Arjuna)

*Terminalia arjuna*is aneffective cardioprotective agent (inducedheat shock protein in myocardium) Maulik et al. used fora long time [225]. Based on the experiences of centuries, the decoction of arjuna bark is used in the Indian sub continental areas for the treatment of congestive heart failure, dyslipidemia, anginal pain and hypertension. The different phytoconstituents present are flavonoids, triterpenoids, β-sitosterol, glycosides [226], arjunetosides I–IV, arjunone, arjunine, arjunolone, saponnins, arjunetein, oligomericproanthocyanidins, leteilin, ellagic acid, phytosterols, gallic acid, arjungenin, arjunic acid, tannins, arjunolic acid and minerals [227].

*Arjuna* has prostaglandin E2-like action through hypotension and coronaryvasodilatation in myocardial ischemia induced by isoprenaline. The bark extract can also decrease oxidative stress induced by isoprenaline [226]. The key advantage of *Terminalia arjuna* is to improve cardiac muscle activity followed by enhanced pumping function of the heart. It is reported that the inotropic effect of *Terminaliaarjuna*might be because of saponin glycosides while vascular strengthening and antioxidant action were owing to OPCs and flavonoids. Cardenolides boost the cardiac contraction force by an increase in both sodium and calcium intracellularly [227]. It also has mild diuretic, cardiotonic anti-inflammatory, ROS scavenging, prostaglandin E2, antithrombotic, antiplatelet effects, anti-atherogenic effects and hypolipidaemicaction. It is also used to treat alone and/or with statin to treat coronary artery disease.All these biological properties make *Terminalia arjuna* aunique medicinal plant currently [228, 229].
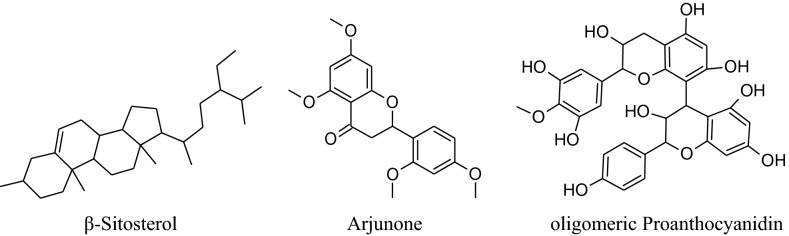



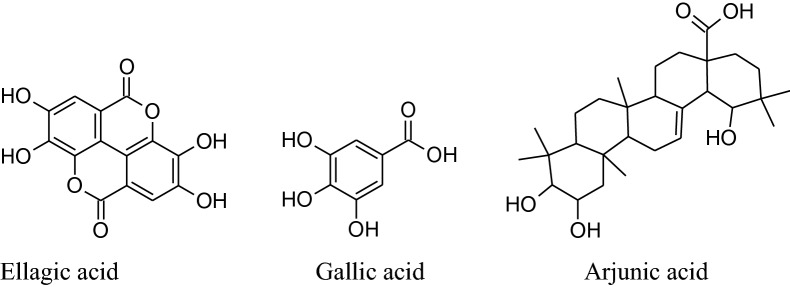


## Conclusion

Nowadays, it is the most important to search the more effective way to treat hypertension and CVD that is the prime cause of death, globally. Nature indeed inspires or produces all new, small chemical entities introduced as a medicine during the decays. Possibly, this is the reason why most patients commonly visit herbal medicine than allopathic for CVD treatment. In this review, we discussed the most commonly used different plants for the management and treatment of hypertension with their mechanism of action. The pharmacological activities of natural plants and their isolates affect the pathogenesis of hypertension by modulating several parameters like endothelial function, ROS production, pro-inflammatory signaling, platelet activation, opening and closing of different ion channels, ACE inhibition, gene expression etc. Surely, the herbal remedies will be of more attention in the coming time, as they possessing a broad spectrum of achievement, after needful clinical and experimental studies. It is also advisable that patients should be properly educated in relation to the consumption of herbs that are used for a long time e.g. black cumin, coriander, garlic, Chinese sage, ginger and ginseng. As some drugs are also available that can raise blood pressure and can be harmful to patients.
